# Comparative morpho-physiological and biochemical responses of *Capsicum annuum* L. plants to multi-walled carbon nanotubes, fullerene C60 and graphene nanoplatelets exposure under water deficit stress

**DOI:** 10.1186/s12870-024-04798-y

**Published:** 2024-02-17

**Authors:** Seyede Zahra Ahmadi, Bahman Zahedi, Mansour Ghorbanpour, Hasan Mumivand

**Affiliations:** 1 Department of Horticultural Sciences, Faculty of Agriculture, Lorestan University, P.O. Box 465, Khorramabad, Iran; 2 Department of Medicinal Plants, Faculty of Agriculture and Natural Resources, Arak University, Arak, 38156-8-8349 Iran; 3 Institute of Nanoscience and Nanotechnology, Arak University, Arak, 38156-8-8349 Iran

**Keywords:** Carbon nanomaterials, Photosynthetic pigments, Gas exchange, Phenol, Antioxidant activity, Drought stress, Toxicity

## Abstract

Water deficit stress is one of the most significant environmental abiotic factors influencing plant growth and metabolism globally. Recently, encouraging outcomes for the use of nanomaterials in agriculture have been shown to reduce the adverse effects of drought stress on plants. The present study aimed to investigate the impact of various carbon nanomaterials (CNMs) on the physiological, morphological, and biochemical characteristics of bell pepper plants subjected to water deficit stress conditions. The study was carried out as a factorial experiment using a completely randomized design (CRD) in three replications with a combination of three factors. The first factor considered was irrigation intensity with three levels [(50%, 75%, and 100% (control) of the field capacity (FC)] moisture. The second factor was the use of carbon nanomaterials [(fullerene C60, multi-walled carbon nanotubes (MWNTs) and graphene nanoplatelets (GNPs)] at various concentrations [(control (0), 100, 200, and 1000 mg/L)]. The study confirmed the foliar uptake of CNMs using the Scanning Electron Microscopy (SEM) technique. The effects of the CNMs were observed in a dose-dependent manner, with both stimulatory and toxicity effects being observed. The results revealed that exposure to MWNTs (1000 mg/L) under well-watered irrigation, and GNPs treatment (1000 mg/L) under severe drought stress (50% FC) significantly (*P* < 0.01) improved fruit production and fruit dry weight by 76.2 and 73.2% as compared to the control, respectively. Also, a significant decrease (65.9%) in leaf relative water content was obtained in plants subjected to soil moisture of 50% FC over the control. Treatment with GNPs at 1000 mg/L under 50% FC increased electrolyte leakage index (83.6%) compared to control. Foliar applied MWNTs enhanced the leaf gas exchange, photosynthesis rate, and chlorophyll a and b concentrations, though decreased the oxidative shock in leaves which was demonstrated by the diminished electrolyte leakage index and upgrade in relative water content and antioxidant capacity compared to the control. Plants exposed to fullerene C60 at 100 and 1000 mg/L under soil moisture of 100 and 75% FC significantly increased total flavonoids and phenols content by 63.1 and 90.9%, respectively, as compared to the control. A significant increase (184.3%) in antioxidant activity (FRAP) was observed in plants exposed to 200 mg/L MWCNTs under irrigation of 75% FC relative to the control. The outcomes proposed that CNMs could differentially improve the plant and fruit characteristics of bell pepper under dry conditions, however, the levels of changes varied among CNMs concentrations. Therefore, both stimulatory and toxicity effects of employed CNMs were observed in a dose-dependent manner. The study concludes that the use of appropriate (type/dose) CNMs through foliar application is a practical tool for controlling the water shortage stress in bell pepper. These findings will provide the basis for more research on CNMs-plant interactions, and with help to ensure their safe and sustainable use within the agricultural chains.

## Introduction

Bell pepper (*Capsicum annuum* L.) is a valuable fruit vegetable from the Solanaceae family. It is the world's third most-produced summer crop after potatoes and tomatoes [1]. The plant is an annual with short branches and oval or egg-shaped, uncut leaves. Its main root is long, up to 80 cm deep, and has a weak ability to produce adventitious roots, meaning that deep planting of seedlings is useless [2]. Bell pepper blooms in late spring to early summer, with white, yellow, light green, purple, and red colored flowers. Its fruit is a berry botanically [3]. Bell pepper fruit can be harvested at the stage of physiological maturity [4]. It is loved by consumers worldwide due to its excellent taste and nutritional value [5]. Bell pepper is high in antioxidants, vitamin C, carotenoids, phenolic compounds (especially flavonoids), and potassium, making it a nutritious household staple [6].

Water scarcity is expected to cause a 30% increase in drought severity worldwide by 2100 due to global warming [7]. Abiotic stresses in agriculture, exacerbated by climate change, cause significant yield losses [8]. Agriculture is the largest consumer of water globally, with 70% of withdrawals occurring in developed countries and 95% in developing countries [9]. Water stress is the most dominant abiotic factor affecting plant growth and development, impacting the performance of plants worldwide [10, 11]. Increasing agricultural productivity by eliminating drought will be a significant challenge in the coming years. Drought stress causes a decrease in growth, physiological and biochemical traits in two Ethiopian red pepper cultivars, with the negative effects more noticeable in the local variety than the Markofana cultivar [12]. Developing plant species that can tolerate drought stress is crucial for agriculture. Studies show that ascorbic acid can enhance drought stress tolerance in peppers by reducing the negative effects of drought stress, such as a decrease in fruit number, plant height, yield, and chlorophyll content. Drought stress also increases the activity of certain enzymes and compounds, such as antioxidant enzymes, compatible solutes, anthocyanins, malondialdehyde (MDA), and hydrogen peroxide (H_2_O_2_) contents in pepper leaves [13].

The use of carbon nanoparticles in agriculture and environmental applications, as well as the possibility of accidental release, can have a significant impact on living organisms, especially plants. Plants are a vital part of both natural and agricultural ecosystems as they are a crucial component of food chains. Interestingly, some nanoparticles possess unique physicochemical properties that can enhance plant growth and stress tolerance. Instead of acting as carriers, these nanoparticles play a biological role that is dependent on their physicochemical properties, concentration, and application method (such as foliar application, hydroponics, and soil drenching) [14]. Zhao et al. [15] have demonstrated the significance of these factors in determining the effectiveness of nanoparticles in boosting plant growth and stress tolerance. Engineered nanomaterials have exhibited promising outcomes in combating the harmful impacts of drought stress in plants [16]. Carbon nanomaterials (CNMs) have been found to increase plant photosynthesis, crop growth, and water absorption [17]. They also increase the efficiency of using N, P, and K and the level of antioxidants [18, 19]. SEM images have confirmed the absorption and distribution of fullerene C60 by the leaf system with foliar spraying of two genotypes of chamomile [20]. Combined treatments of compost, *Arbuscular mycorrhizal* fungi, and CNMs have also been found to improve the growth of corn plants and increase soil fertility in both control and drought stress conditions [21]. In investigating the effect of CNMs on chili pepper plants under drought stress, functionalized CNMs were found to increase relative water content (RWC), chlorophyll fluorescence parameter (*Fv/Fm*) and chlorophyll stability index, while decreasing abscisic acid content in the leaves. Exogenous application of functionalized CNPs also increased the activity of antioxidant enzymes such as superoxide dismutase and catalase [22].

Drought is a significant abiotic stress that can adversely affect crop yield. Carbon nanoparticles have shown potential in enhancing plant growth and productivity under abiotic stress conditions. However, the impact of using carbon nanoparticles on bell pepper (*C. annuum* L.) has not been evaluated yet. The present study aimed to assess the impacts of CNMs [(fullerene C60, multi-walled nanotubes (MWCNTs) and graphene nanoplatelets (GNPs)] on the morpho-physiological and biochemical features of bell pepper under drought stress environment. Developing appropriate tactics or treatments to improve plant tolerance to water stress can benefit from such information.

## Materials and methods

### Plant materials, growth conditions and treatments

The current research was carried out as a factorial experiment in the form of completely randomized block design (CRBD) with three replications (*n* = 3) in the greenhouse of the Faculty of Agriculture of Lorestan University. The first factor was included irrigation intensities at three levels [(50%, 75%, and 100% (control) of the field capacity (FC) moisture)], and the second factor was designated the use of carbon nanomaterials (CNMs) [(fullerene C60, multi-walled carbon nanotubes (MWNTs) and graphene nanoplatelets (GNPs)] at different concentrations [(control (0), 100, 200, and 1000 mg/L). Five bell pepper seeds were planted in each pot. After germination of the seeds, only one seedling was kept and the other seedlings were removed. The diameter of the opening of the pots used for the research was 20 cm and its height was 30 cm, which were filled with 10 kg of culture medium. The substrate prepared for the pots was composed of field soil, sand, manure in a ratio of 1:1:1. To prepare a stock solution of CNMs, 0.55 g of each nanoparticle was poured into 50 mL of distilled water for 30 min and ultrasonicated (4-L ultrasonic bath model Zealway (Xiamen), China) so that the CNMs do not clump in the water and are completely distributed. Then, it was made up to 500 mL with distilled water and different concentrations of CNMs were prepared from the stock solution. The first spraying of different doses of CNMs was done at the four-leaf stage and the second spraying was done two weeks later. Two days after the second foliar spraying, water deficit stress treatments were started and continued one week before harvesting. In order to determine the value of FC, the pots were first weighed and then irrigated. Plastic was placed on the pots and 24 h later (after gravity water exit) the pots were weighed. The difference in their weight indicated the amount of water available to the plant, that is the FC. The characteristics of the soil of the pots are presented in Table 1. Seeds were purchased from Keshtzar Company (Tehran) and CNMs were purchased from Iranian Nano Materials Pioneers Company (Mashhad) to perform the experiment. The specific characteristics of applied CNMs are given in Table 2.

**Table 1:** Soil physical and chemical characteristics in this study

Texture	EC	pH	O.C	N	P	K
	(dS m^−1^)		(%)		mg kg^−1^	
Sandy clay loam	2.45	6.9	1.2	0.17	14.85	367

**Table 2:** Characteristics of carbon nanomaterials used in this study

Fullerene C60	Morphology	Color	Decoloration rate	Purity	Sterilization	APS	H_2_O	Ash	pH	True density	Bulk density
	Nanospherical	Black	99.00%	> 95%	Cobalt-60 Radiation	20–40 nm	< 5%	< 2%	7–10	0.44 g/mL	0.32 g/mL
Multi-walled carbon nanotubes (MWNTs)	Morphology	Color	Outside diameter	Purity	Inside diameter	SSA	Length	Ash	EC	True density	Bulk density
	nanotube	Black	20–30 nm	> 95%	5–10 nm	> 110 m^2^/g	10–30 um	< 1.5%	> 100 s/cm	~ 2.1 g/cm^3^	0.28 g/cm^3^
Graphene Nanoplatelets (GNPs)	Morphology	Color	Volume Resistivity	Purity	diameter	SSA	Thickness	The Product COA	pH	True density	Bulk density
	Nanoplatelets Powder	Black	4 × 10^–4^ Ω.cm	99.50%	4 -12 um	500 -1200 m^2^/g	2–18 nm, < 32 layers	C = 99.7%, O < 0.3%	7–7.7	-	-

### Morphological traits measurement

At the end of the experiment, the height of the plant was determined by a ruler. Next, the number of flowers was counted. Also, fresh weight of the plant was measured with a digital scale of 0.001 g. Then, to measure the dry weight of the plant, it was placed in an oven at 70 °C for 48 h and its dry weight was also calculated. To determine the fresh weight and dry weight of the roots, first remove the roots from the soil in such a way that they are not damaged and washed the flowers attached to the roots with water and after drying the surface moisture, we weighed the samples. To determine dry weight, the root samples were kept in an oven at 70 °C for 48 h.

### Agronomic traits

About 132 days after seed germination, the fruits from each plant were picked and counted separately. A digital scale was used to determine the fresh weight and dry weight of the fruits. After measuring the fresh weight and in order to measure the dry weight, the fruits were placed in an oven at 70 °C for 48 h. The length and diameter of the fruit were recorded using a digital caliper.

### Physiological traits

#### Leaf relative water content

In order to measure RWC of the leaf, sampling was done from the last fully developed leaf of all the experimental treatments at 8:00 am and the weight of the samples was calculated in the laboratory with an accuracy of 0.001 g, and then all the obtained samples were placed in double- distilled water and was maintained at room temperature (~ 25 °C) for 24 h. Then, the saturated weight of the leaves was recorded, and the leaves were placed in the oven at 70 °C for another 24 h and the dry weight of each was determined. By putting the numbers obtained from weighing in the following formula, the relative content of leaf water was calculated [23].

$$\mathrm{RWC\ }(\mathrm{\%}) = ({\text{FW}}-{\text{DW}}) / ({\text{SW}}-{\text{DW}}) \times 100$$

where, FW, DW and SW are fresh, dry and saturated weights of the leaf sample, respectively.

#### Electrolyte leakage index

In order to estimate the stability of the cell membrane in leaves, the measurement of their electrolyte leakage is used. For this purpose, identical circles were prepared from the fully developed leaves of each treatment. The experiment involved placing pieces of leaves from different treatments in a glass tube filled with distilled water and leaving them at room temperature for 24 h. The electrical conductivity (EC1) of the solution was measured after this time. Next, the tubes were put in an autoclave at 120 °C for 20 min to investigate the electrolyte leakage of dead cells. After cooling, the electrical conductivity of the solution (EC2) was calculated again. The percentage of electrolyte leakage (EL) from the membranes was determined using the following equation [24].

$$\mathrm{EL\ }(\mathrm{\%}) = ({\text{EC}}1/{\text{EC}}2) \times 100$$

#### Gas exchanges

Gas exchange factors were measured in the upper leaves using a portable gas exchange measurement device model CI-340 CID, made by USA. At the time of gas exchange measurement, the carbon dioxide under the aperture was 350 μmol/mol, the temperature under the chamber was 29–26 °C, and the relative humidity was 58–62%. The work of this device is based on the amount of carbon dioxide consumed. Stomatal conductance was measured based on μmol H_2_O/m^2^ s and photosynthesis rate was measured based on μmol CO_2_/m^2^ s.

### Biochemical traits

#### Chlorophyll and carotenoids content

The amount of chlorophyll was calculated by the protocol of Arnon [25] and carotenoids by the method of Lichtenthaler and Wellburn [26]. For this purpose, 0.5 g of fresh leaf sample was extracted after weighing in a Chinese mortar with 10 mL of 80% acetone. Then the obtained extract was centrifuged for 10 min at 3000 rpm. Then, 3 mL of the supernatant solution was poured into the spectrophotometer (speco 200 model spectrophotometer manufactured by Analyticjena, Germany) and the optical absorption of chlorophyll a, chlorophyll b and total carotenoids was read at 663, 645 and 470 nm wavelengths, respectively. Using the following formulas, the concentration of chlorophyll a, chlorophyll b and total carotenoids (xanthophyll and carotene) was calculated in terms of mg g^−1^ fresh weight (FW).

$$\begin{array}{l}\mathrm{Chlorophyll\ a }= 12.7({{\text{A}}}_{663}) - 2.69({{\text{A}}}_{645})\\ \mathrm{Chlorophyll b\ }= 22.9({{\text{A}}}_{645}) - 4.68({{\text{A}}}_{663})\\ \mathrm{Total\ carotenoids }= [1000({{\text{A}}}_{470}) - 2.27 \times \mathrm{ Chl a }-81.4 (\mathrm{Chl b})]/229\end{array}$$

#### Total phenols and flavonoids

To prepare the sample, 1 g of dried leaves was ground into powder and mixed with 10 mL of 80% methanol. The mixture was then placed in an ultrasonic bath for 30 min. Afterward, the sample was centrifuged at 14,000 rpm for 10 min. The resulting supernatant solution was used to measure the total phenol, total flavonoids, and antioxidant properties. The content of total phenol was determined using the Folin-Ciocalteu protocol [27]. According to this method, 100 µl of the extract with a concentration of 1 mg/mL was added to 500 µL of Folin's reagent and after 1 min, 1.5 mL of 20% sodium bicarbonate was added to each tube and then vortexed and it is incubated for 120 min at room temperature. Thereafter, the absorbance of the sample at 760 nm was read by a spectroscopic device. The standard curve was then prepared by solutions of 50 to 500 mg/L of gallic acid in methanol (*R*^*2*^ = 0.997, y = 0.003x + 0.0868). Total phenol content was expressed as mg gallic acid equivalent/g DW, which is a reference compound for determining phenol content.

The value of flavonoids present in the extracts was assessed using the aluminum chloride colorimetric method [28]. For this, 0.5 mL of each extract was mixed with 1.5 mL of methanol and 0.1 mL of 10% aluminum chloride. Following this, 1.10 mL of 1 M potassium acetate and 2.8 mL of distilled water were added to the mixture, which was then incubated at room temperature for 20 min. Finally, the absorbance of the mixture was recorded at 415 nm using a spectroscopic device. Different concentrations of rutin 12.5–100 µg/mL in methanol were used to draw a standard curve (*R*^*2*^ = 0.965, y = 0.0054x + 0.1746) and the content of the extract was expressed as mg Rutin equivalents/g DW.

#### Evaluation of antioxidant properties

Determining the antioxidant activity of the obtained extracts by FRAP (Ferric Reducing Antioxidant Potential): In this method, antioxidants that have the ability to regenerate Fe^3+^ to Fe^2+^, cause the colorless TPTZ-Fe^3+^ complex to become TPTZ-Fe^2+^ complex, which is blue in color and Its intensity can be measured at the wavelength of 593 nm. For this purpose, the concentration of 250 µg/ mL of the plant extract was taken and added to the final volume of 2 mL of FRAP solution containing 10 mM TPTZ (in 40 mM HCl), 20 mM ferric chloride and 300 mM acetate buffer at pH = 3.6 became. The above sample was kept at a temperature of 37 °C for 10 min and the intensity of the resulting color was noted at 593 nm against a blank. To draw the standard curve for the FRAP method, ferrous sulfate (FeSO_4_, 7H_2_O) with concentrations of 1000, 500, 250, 125 µM was used (*R*^*2*^ = 0.976, y = 0.0025x—0.0394) and the antioxidant power of the extracts was based on the Fe^2+^ µmol/g of dry weight (DW) [29].

### SEM observations

The samples were prepared based on the protocol described by Rao and Shekhawat (2014) [30]. To do this, the samples were fixed in a solution containing 2.5% v/v glutaraldehyde and potassium phosphate buffer (0.05 M, pH 7.1) for 8 h. After that, the samples were gently dehydrated using graded series of ethanol (10%-70%) for 20 min at each step. Finally, dehydrated samples were sputter-coated with a gold layer using an ion sprayer, and the leaf surface morphology was then analyzed by using SEM device (JEOL, Japan).

### Statistical analysis

Analysis of the obtained data was carried out by using SAS software (Ver.9.1), and comparison of treatment averages/significance within the means was performed using Duncan's test (DMRT) at the 5% probability level. ANOVA assumptions were examined by using Shapiro–Wilk test for normality, and Levene´s test for homogeneity of variance among the variables. The graphs were drawn in MS-Excel. The data represented in this study are means (± SD) of three (*n* = 3) biological replicates.

## Results

Analysis of variance (ANOVA, Table 3) showed that the impact of water stress on ion leakage, chlorophyll a and carotenoids contents had no significant difference (*P* ˃ 0.05), but on other traits evaluated in this study, it had a significant effect at the probability level of 1%. Also, foliar application of CNMs had a significant effect (*P* ≤ 0.05) on the examined traits except for height, fresh and dry weight of shoots, fresh weight of roots, relative leaf water content, ion leakage and chlorophyll and carotenoids content. The interaction effect of the treatments on height, fresh and dry weight of shoot, relative content of leaf water, chlorophyll a and carotenoids did not show any significant effect. While the interaction effect of the treatments had a significant effect on the ion leakage trait at the 5% probability level and on other traits at the 1% probability level.

**Table 3:** Analysis of Variance (ANOVA) of the effect of foliar application of carbon nanomaterials and water deficit stress on morpho-physiological and phytochemical characteristics of bell pepper plant

S.O.V	d.f	Plant height	Number of flowers	Fresh weight of shoot	Dry weight of shoot	Fresh weight of root	Dry weight of root
Block	2	95.59^ns^	5.14^ns^	40.81^ns^	1.72^ns^	904.35^b^	0.55^ns^
Water deficit stress (A)	2	2481.74^a^	182.63^a^	11,767.72^a^	407.35^a^	2007.91^a^	31.10^a^
Carbon nanomaterials (B)	9	76.01^ns^	26.01^a^	276.89^ns^	6.04^ns^	278.44^ns^	30.54^a^
A × B	18	55.01^ns^	23.56^a^	162.59^ns^	5.36^ns^	400.76^a^	25.55^a^
Error	57	68.61	5.94	153.22	4.58	157.27	2.57
CV	-	15.58	33.42	17.97	17.00	18.05	15.10
S.O.V	d.f	Number of fruits	Fresh weight of fruit	Dry weight of fruit	Length of fruit	Fruit diameter	Relative water content
Block	2	0.31^ns^	7.24^ns^	0.07^ns^	61.50^ns^	114.61^b^	78.72^b^
Water deficit stress (A)	2	53.15^a^	67.77^a^	1.00^a^	1211.27^a^	1158.75^a^	144.14^a^
Carbon nanomaterials (B)	9	2.03^a^	22.37^a^	0.50^a^	286.66^a^	313.24^a^	31.66^ns^
A × B	18	3.55^a^	48.02^a^	0.86^a^	522.14^a^	406.83^a^	34.74^ns^
Error	57	0.51	7.22	0.12	46.67	34.82	24.25
CV	-	24.75	33.93	29.98	16.05	16.83	7.23
S.O.V	d.f	Ion leakage index	Photosynthesis rate	Stomatal conductance	Chlorophyll a	Chlorophyll b	Carotenoid
Block	2	365.56^ns^	22.33^ns^	11,142.49^ns^	0.01^ns^	0.003^ns^	0.003^ns^
Water deficit stress (A)	2	590.20^ns^	1216.55^a^	14,870.53^a^	0.002^ns^	0.31^a^	0.003^ns^
Carbon nanomaterials (B)	9	134.92^ns^	111.24^a^	22,341.28^a^	0.007^ns^	0.11^a^	0.001^ns^
A × B	18	409.26^b^	113.19^a^	10,108.22^a^	0.006^ns^	0.04^a^	0.001^ns^
Error	57	197.71	15.76	1057.61	0.009^ns^	0.006	0.001
CV	-	23.59	20.45	11.62	16.77	14.65	17.83
S.O.V	d.f	Total phenol	Total flavonoids		FRAP		
Block	2	0.08^ns^	0.03^ns^		0.002^ns^		
Water deficit stress (A)	2	0.65^a^	1.19^a^		0.19^a^		
Carbon nanomaterials (B)	9	0.52^a^	0.45^a^		0.13^a^		
A × B	18	0.98^a^	0.35^a^		0.09^a^		
Error	57	0.06	0.11		0.02		
CV	-	16.18	23.28		29.03		

^a^Significant at 1% level. ^b^Significant at 5%, ^ns^ not statistically significant. *CV* Coefficient of variation

### Plant height and number of flowers per plant

**Fig. 1:**
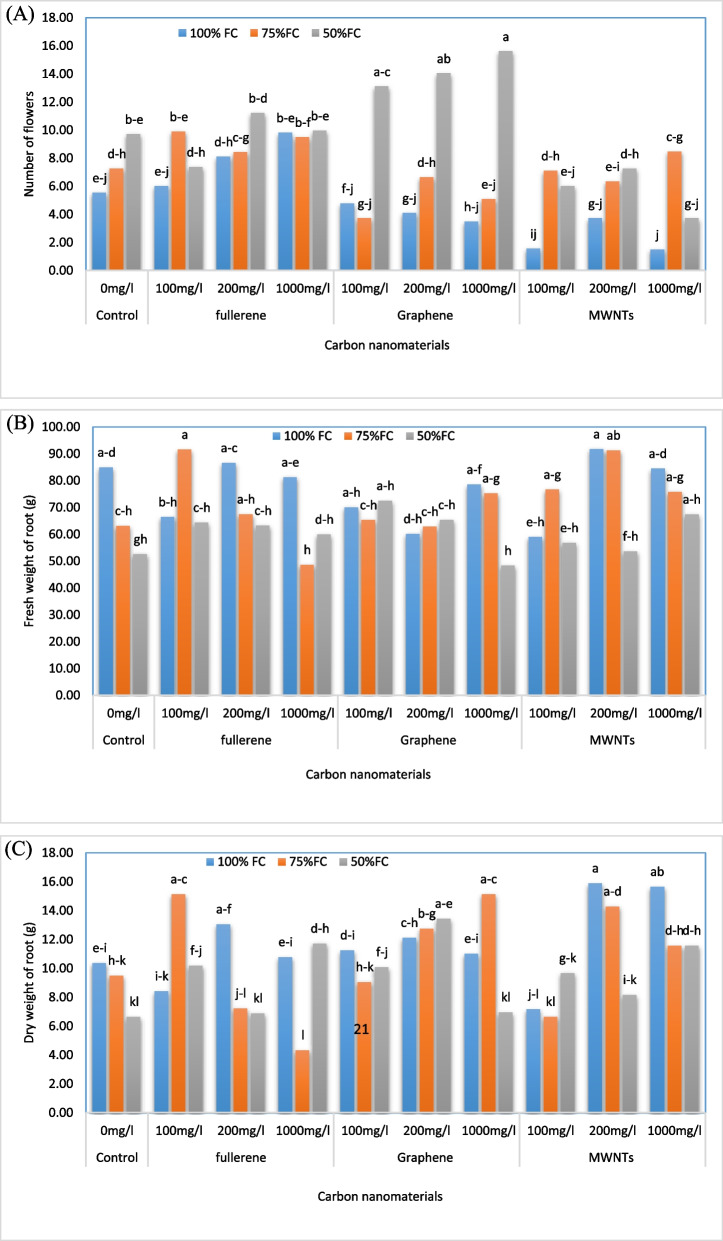
Change in the number of flowers (**A**), fresh weight (**B**) and dry weight (**C**) of roots in bell pepper plants exposed to different types of nanomaterials (fullerene C60, multi-walled carbon nanotubes and graphene nanoplatelets) at different concentrations (0, 100, 200 and 1000 mg/L) under drought stress intensities [(50%, 75%, and 100% (control) of field capacity (FC) moisture)]. The values reported are means ± SD (*n* = 3) and the Bars with different letters show significant difference among employed treatments at *P* < 0.05 probability level using Duncan’s test

CNMs played a major share in plant height and number of flowers per plant under water deficit conditions, when analyzed with control groups. Mean comparison of the individual effects of drought stress on plant height is presented in Table 4. It was observed that the highest plant height (60.19 cm) was related to irrigating the plants with 100% FC and the lowest plant height (88/42 cm) was observed when the plants were irrigated with 50% FC. The results of comparing the mean interaction effect of the treatments showed that the highest number of flowers (Fig. 1A) (15.59 flowers) was related to the application of 1000 mg/L of GNPs at the irrigation level of 50% FC. This was 182% higher than the control. On the other hand, the lowest number of flowers (1.49 flowers) was related to the application of 1000 mg/L of MWNTs and the irrigation level of 100% FC, which showed a 73% decrease compared to the control.

**Table 4:** The comparison of the mean effect of water deficit stress treatments and carbon nanomaterials foliar application on the morpho-physiological and phytochemical characteristics of bell pepper plants

Treatment	Treatment concentration/level	Plant height (cm)	Fresh weight of shoot (g)	Dry weight of shoot (g)	RWC (%)	Chlorophyll a (mg g^−1^ FW)	Carotenoid (mg g^−1^ FW)
Water deficit stress	100% FC	60.19a	87.62a	15.85a	70.37a	0.57a	0.19a
	75% FC	56.53a	70.96b	13.34b	68.09ab	0.56a	0.19a
	50% FC	42.88c	48.16c	8.59c	65.98b	0.55a	0.21a
Carbon nanomaterial	Control	50.94a	67.03a	11.98a	7.87a	0.55a	0.20a
	Graphene (100 mg/L)	54.00a	67.23a	12.31a	65.33a	0.52a	0.19a
	Graphene (200 mg/L)	51.22a	67.14a	12.65a	68.05a	0.55a	0.19a
	Graphene (1000 mg/L)	56.67a	76.09a	12.06a	65.80a	0.57a	0.20a
	Fullerene (100 mg/L)	56.67a	75.69a	14.62a	66.94a	0.60a	0.19a
	Fullerene (200 mg/L)	56.56a	75.86a	12.48a	69.90a	0.52a	0.20a
	Fullerene (1000 mg/L)	54.89a	70.01a	12.78a	68.75a	0.56a	0.21a
	MWNTs (100 mg/L)	49.13a	59.57a	11.35a	71.00a	0.54a	0.21a
	MWNTs (200 mg/L)	49.11a	68.13a	12.25a	68.08a	0.59a	0.21a
	MWNTs (1000 mg/L)	52.00a	61.12a	12.19a	67.07a	0.60a	0.20a

Means with the same letters in columns do not have a significant difference at the 1% probability level based on the Duncan test

*Abbreviations*: Fullerene (fullerene C60), *MWNTs* multi-walled carbon nanotubes, *GNPs* graphene nanoplatelets, *RWC* relative water content, *FC* field capacity

### Fresh and dry weight of shoots and roots

According to the results (Table 4, Fig. 1B, C), the treatment of water deficit stress had the highest fresh and dry weight of shoots when the irrigation level was at 100% FC, which corresponded to 87.62 g and 15.85 g, respectively. The lowest wet and dry weight of shoots occurred when the irrigation level was at 50% FC, which corresponded to 16.48 g and 59.8 g, respectively. In terms of the characteristics of fresh and dry weight of the root, the comparison of the mean interaction effect of the treatments (Fig. 1B, C) revealed that the highest fresh weight of the root was obtained by applying a concentration of 200 mg/L of MWNTs and irrigating at 100% FC, which resulted in 91.76 g (an increase of 8.02% compared to the control). This concentration of multi-walled nanotubes was not significantly different from the application of 100 mg/L of nano fullerene and irrigation of 75% FC. On the other hand, the lowest fresh weight of the root was obtained by applying a concentration of 1000 mg/L of CNPs at the irrigation level of 50% FC, which resulted in 48.38 g (a decrease of 43.05% compared to the control). Similarly, the highest root dry weight was obtained by applying a concentration of 200 mg/L of MWNTs and irrigating at 100% FC, which resulted in 15.90 g (an increase of 53% compared to the control). The lowest root dry weight was obtained by applying a concentration of 1000 mg/L of nano fullerene at the irrigation level of 75% FC, which resulted in 4.34 g (a decrease of 58.2% compared to the control).

### Number of fruits, fresh and dry weights of fruit

**Fig. 2:**
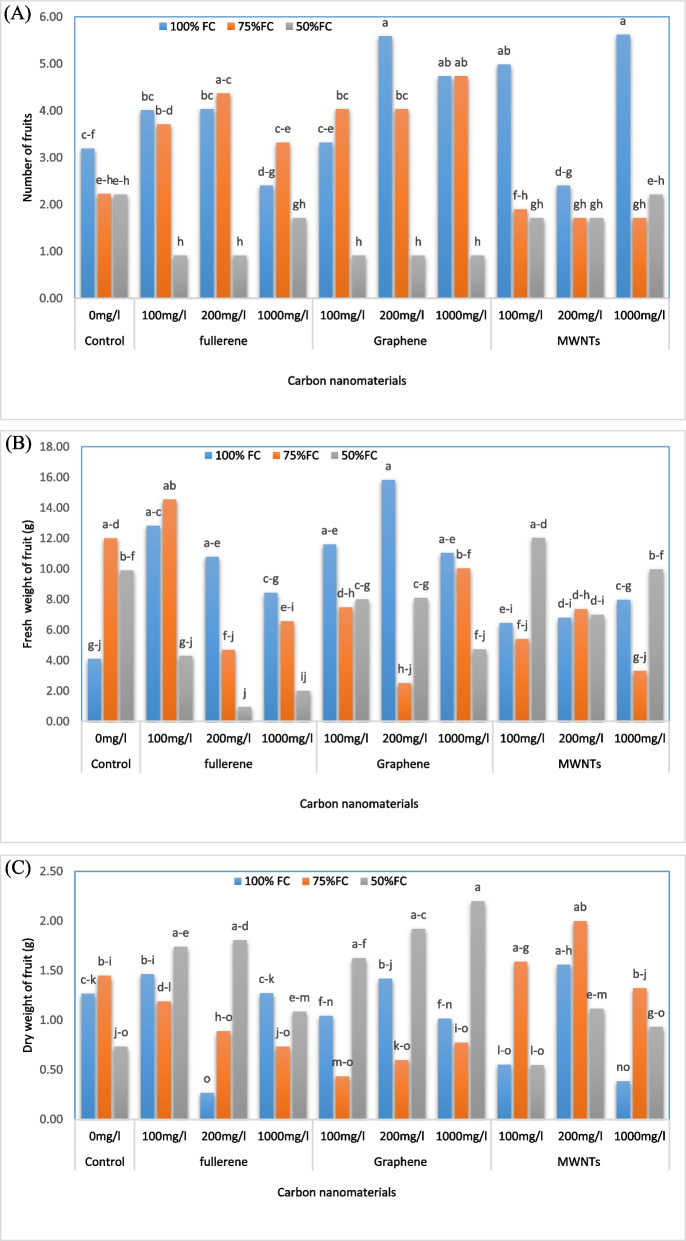
Change in the number of fruits (**A**), fresh weight (**B**) and dry weight (**C**) of fruits in bell pepper plants exposed to different types of nanomaterials (fullerene C60, multi-walled carbon nanotubes and graphene nanoplatelets) at different concentrations (0, 100, 200 and 1000 mg/L) under drought stress intensities [(50%, 75%, and 100% (control) of field capacity (FC) moisture)]. The values reported are means ± SD (*n* = 3) and the Bars with different letters show significant difference among employed treatments at *P* < 0.05 probability level using Duncan’s test

It has been observed that the highest number of fruit (5.62) was obtained with the application of 1000 mg/L of MWNTs and irrigation at 100% field capacity (FC) (Fig. 2A). This resulted in a 76.17% increase in fruit production compared to the control. This treatment was not significantly different from the application of 200 mg/L of GNPs at the same level of irrigation. Additionally, the study compared the effects of different levels of irrigation and carbon nanoparticle foliar spraying on the fresh and dry weight of the fruit (Fig. 2B, C). It was observed that the highest fresh weight (15.81 g) was obtained with the treatment of 200 mg/L of GNPs and irrigation at 100% FC, resulting in a 284.67% increase compared to the control. The highest dry weight (2.20 g) was obtained with the treatment of 1000 mg/L of GNPs and irrigation at 50% FC, resulting in a 73.23% increase compared to the control. On the other hand, the lowest fresh and dry weight were observed with the application of 200 mg/L of nanofullerene and irrigation at 50% FC, resulting in a 76.89% reduction and with the application of 200 mg/L of nanofullerene and irrigation at 100% FC, resulting in a 74.78% decrease compared to the control, respectively.

### Fruit length and diameter

**Fig. 3:**
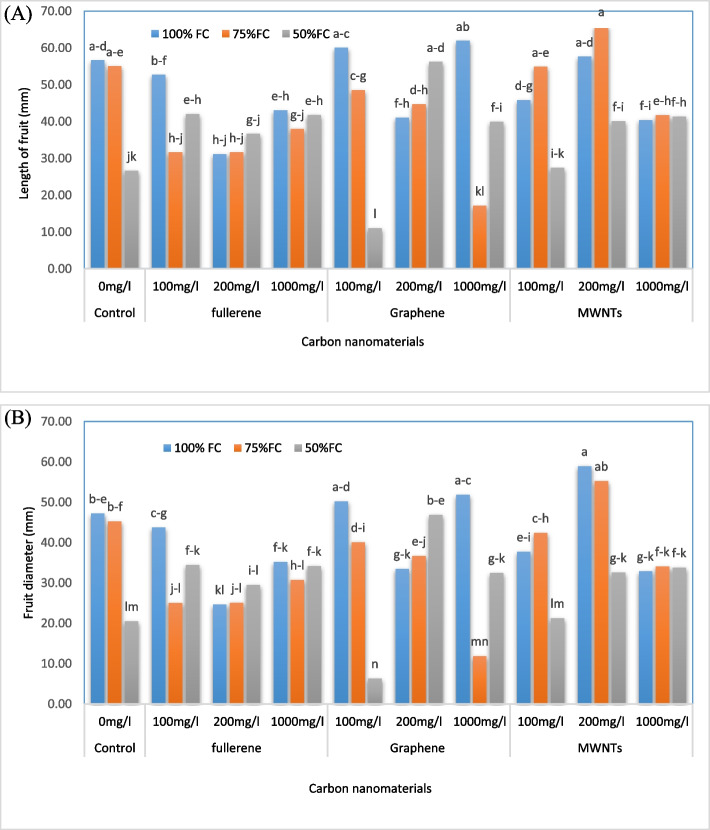
Change in the length (**A**) and diameter (**B**) of fruits in bell pepper plants exposed to different types of nanomaterials (fullerene C60, multi-walled carbon nanotubes and graphene nanoplatelets) at different concentrations (0, 100, 200 and 1000 mg/L) under drought stress intensities [(50%, 75%, and 100% (control) of field capacity (FC) moisture)]. The values reported are means ± SD (*n* = 3) and the Bars with different letters show significant difference among employed treatments at *P* < 0.05 probability level using Duncan’s test

We observed that the treatment of 200 mg/L of multi-walled nanotubes and irrigation of 75% FC resulted in the maximum fruit length of 65.86 mm (16.5% increase compared to the control) (Fig. 3A, B). Similarly, the highest fruit diameter of 58.93 mm (24.85% increase compared to the control) was observed in the treatment of 200 mg/L of MWNTs with an irrigation level of 100% FC. However, the lowest fruit length and diameter were related to the application of 100 mg/L of GNPs and the irrigation level of 50% FC, which resulted in 10.97 mm and 6.34 mm, respectively (80.59% and 86.57% reduction compared to the control, respectively).

### RWC and EL

**Fig. 4:**
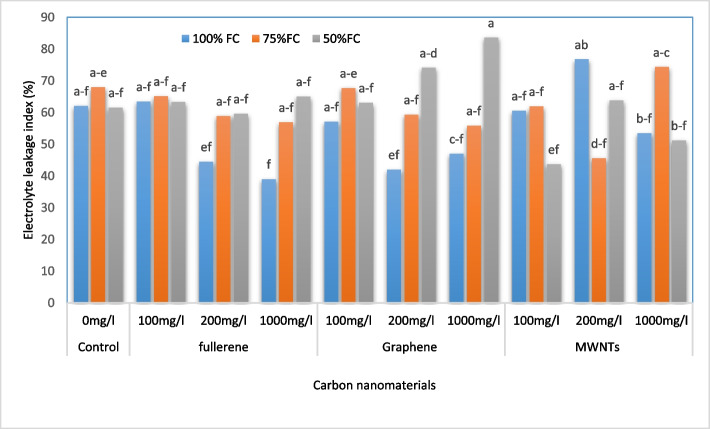
Change in the electrolyte leakage index of bell pepper plants exposed to different types of nanomaterials (fullerene C60, multi-walled carbon nanotubes and graphene nanoplatelets) at different concentrations (0, 100, 200 and 1000 mg/L) under drought stress intensities [(50%, 75%, and 100% (control) of field capacity (FC) moisture)]. The values reported are means ± SD (*n* = 3) and the Bars with different letters show significant difference among employed treatments at *P* < 0.05 probability level using Duncan’s test

The analysis of the data from Table 4 shows that the highest value of relative leaf water content was observed for the irrigation level of 100% FC (70.37%), while the lowest value was associated with the irrigation level of 50% FC (65.98%). Additionally, the findings from Fig. 4 indicate that the highest electrolyte leakage rate was observed for the treatment of 1000 mg/L of GNPs and the irrigation level of 50% FC (83.57%), which represents a 34.62% increase compared to the control. On the other hand, the lowest electrolyte leakage rate was observed for the application of 1000 mg/L of nanofullerene and the irrigation level of 100% of the agricultural capacity (39.02%), which represents a 37.15% decrease compared to the control.

### Photosynthesis rate and stomatal conductance

**Fig. 5:**
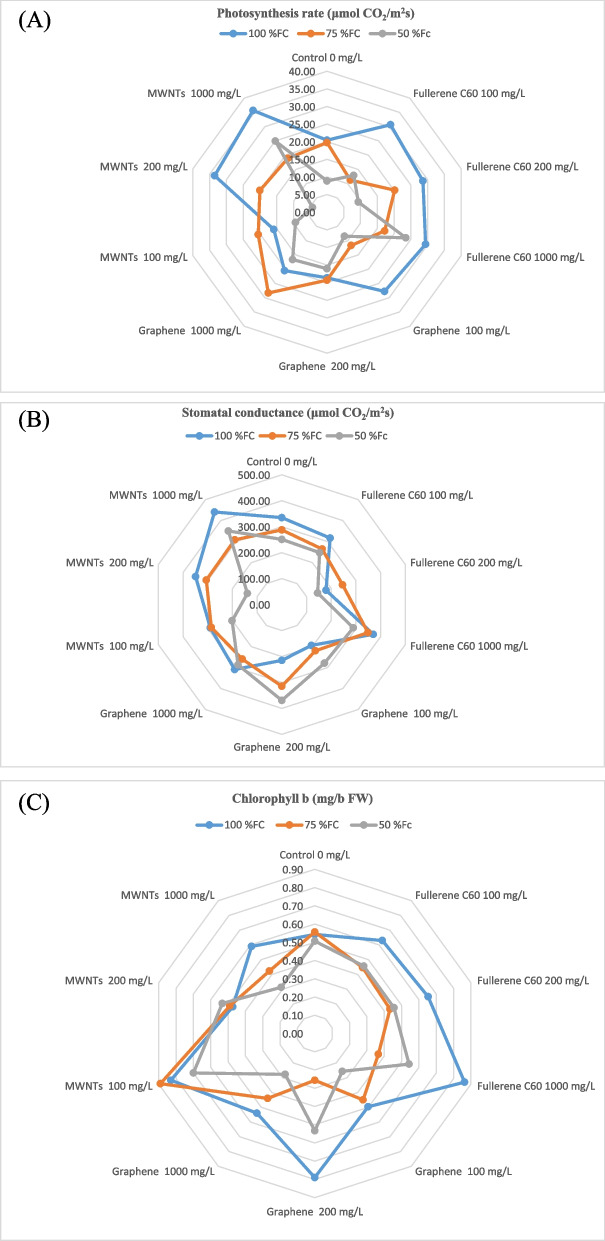
Change in the rate of photosynthesis (**A**), stomatal conductance (**B**), and chlorophyll b (**C**) of bell pepper plants exposed to different types of nanomaterials (fullerene C60, multi-walled carbon nanotubes and graphene nanoplatelets) at different concentrations (0, 100, 200 and 1000 mg/L) under drought stress intensities [(50%, 75%, and 100% (control) of field capacity (FC) moisture)]. The values reported are means ± SD (*n* = 3) and the Bars with different letters show significant difference among employed treatments at *P* < 0.05 probability level using Duncan’s test

In the present study, we found that the highest values of photosynthesis rate and stomatal conductance were obtained when a concentration of 1000 mg/L of multi-walled nanotubes was applied with an irrigation level of 100% FC (Fig. 5A, B). This resulted in 35.68 and 441 μmol CO_2_/m^2^ s, respectively, with an increase of 74.73% and 31.64% compared to the control. However, the lowest values of these traits were obtained when a concentration of 200 mg/L of nanotubes was applied with an irrigation level of 50% FC. This resulted in 4.33 and 139.50 μmol CO_2_/m^2^ s, respectively, which was 78.8% and 58.36% lower than the control.

### Photosynthetic pigments

Results demonstrated that the highest amount of chlorophyll b (0.89 mg/g FW) was found in plants upon irrigation level of 75% FC when 100 mg/L of multi-walled nanotubes were applied (Fig. 5C). This showed an increase of 64.81% compared to the control. However, the lowest amount (0.26 mg g^−1^ FW) was observed at irrigation level of 50% FC when 100 mg/L of GNPs was sprayed on the foliage. This resulted in a decrease of 51.85% compared to the control.

### Total phenols and flavonoids content

**Fig. 6:**
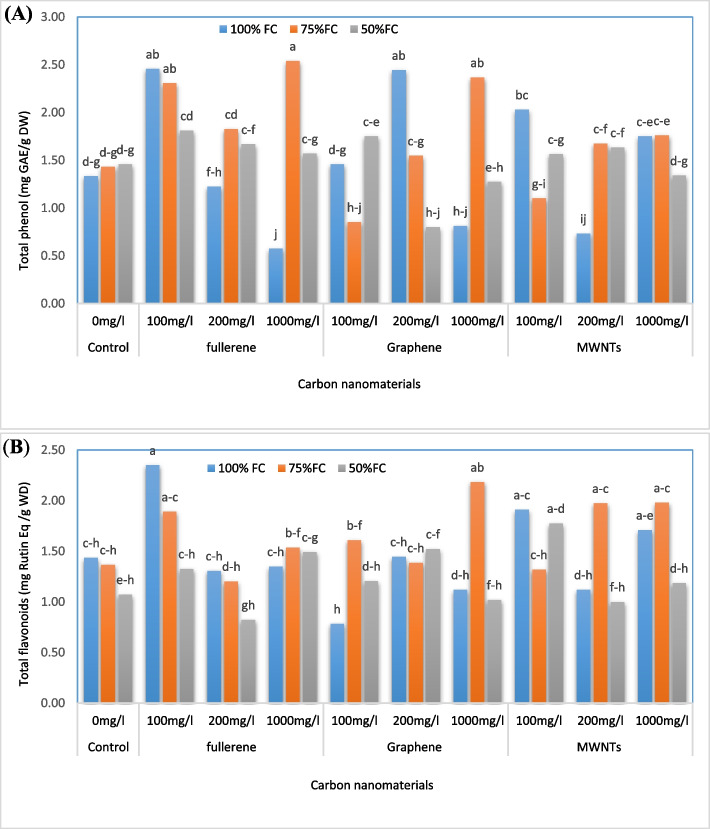
Change in the total phenol (**A**) and flavonoid (**B**) contents of bell pepper plants exposed to different types of nanomaterials (fullerene C60, multi-walled carbon nanotubes and graphene nanoplatelets) at different concentrations (0, 100, 200 and 1000 mg/L) under drought stress intensities [(50%, 75%, and 100% (control) of field capacity (FC) moisture)]. The values reported are means ± SD (*n* = 3) and the Bars with different letters show significant difference among employed treatments at *P* < 0.05 probability level using Duncan’s test

Figure 6 demonstrates that the highest total phenol content (2.54 mg GAE / g DW) was observed when applying 1000 mg/L of nanofullerene with an irrigation level of 75% FC, which resulted in a significant increase of 90.98% compared to the control (Fig. 6A). However, the lowest total phenol content (0.58 mg GAE/g DW) was observed when applying 1000 mg/L of nanofullerene with an irrigation level of 100% FC, which resulted in a significant decrease of 56.39% compared to the control. In addition, the highest total flavonoid content (2.35 mg Rutin Eq/g DW) was observed when applying 100 mg/L of nanofullerene with an irrigation level of 100% FC, which resulted in a significant increase of 63.19% compared to the control. Figure 6 shows that the application of 100 mg/L of GNPs with an irrigation level of 100% resulted in the lowest crop capacity (0.78 mg Rutin Eq/g DW), which was a significant decrease of 45.83% compared to the control (Fig. 6B).

### Antioxidant properties

**Fig. 7:**
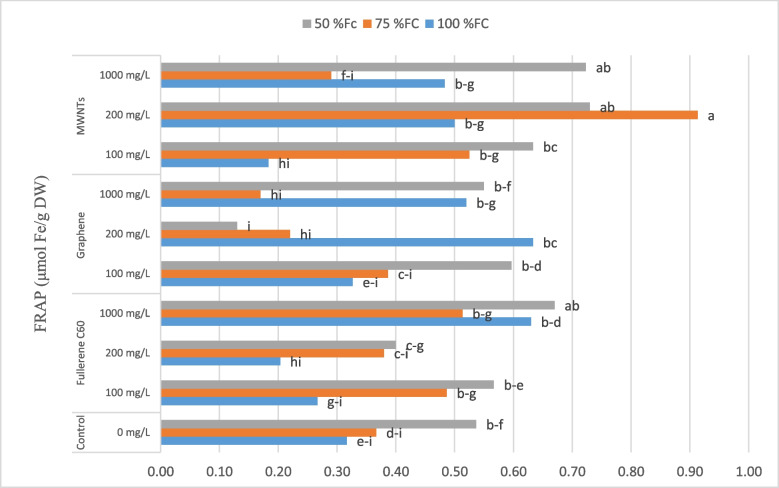
Change in the antioxidant (FRAP) capacity of bell pepper plants exposed to different types of nanomaterials (fullerene C60, multi-walled carbon nanotubes and graphene nanoplatelets) at different concentrations (0, 100, 200 and 1000 mg/L) under drought stress intensities [(50%, 75%, and 100% (control) of field capacity (FC) moisture)]. The values reported are means ± SD (*n* = 3) and the Bars with different letters show significant difference among employed treatments at *P* < 0.05 probability level using Duncan’s test

Mean comparison of the interaction effects among irrigation levels and the use of CNMs treatments on antioxidant activity (Fig. 7) of extracts showed that the highest amount of antioxidant activity (0.91 mmol Fe/g DW) was found in plants treated with 200 mg/L of MWNTs under irrigation of 75% FC. This trait increased by 184.37% compared to the control. However, the lowest amount of antioxidant activity (0.13 mmol Fe/g DW) was observed in plants treated with foliar spraying of 200 mg/L of GNPs and irrigated with 50% FC, which showed a decrease of 59.37% compared to the control.

### SEM images

**Fig. 8:**
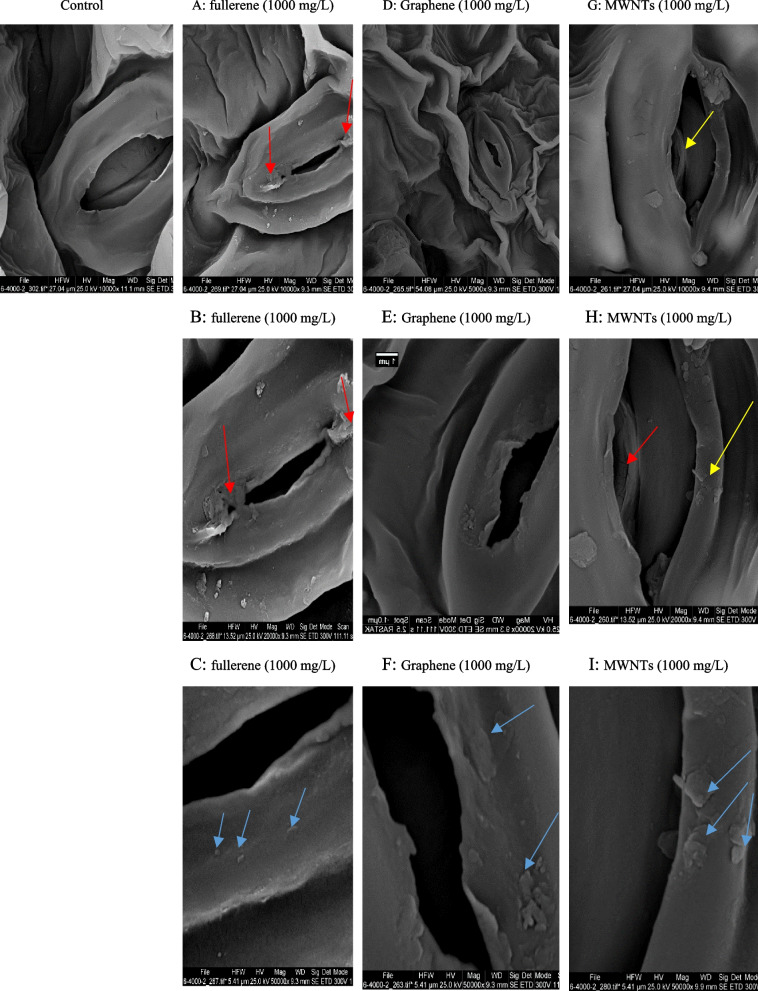
Scanning electron microscope (SEM) images of the untreated control leaf sample (control); leaf sample of C60 fullerene treated (1000 mg/L) (**A**, **B** and **C**); leaf sample of Graphene treated (1000 mg/L) (**D**, **E** and **F**); leaf sample of Multi walled nanotubes treated (1000 mg/L) (**G**, **H** and **I**)

The research conducted on pepper plants treated with CNMs confirmed their uptake and translocation through the leaf system. The confirmation was made using SEM analysis, as shown in Fig. 8, which compares plants treated with CNMs at a concentration of 1000 mg/L and the control group. The SEM images revealed that the MWNTs (Fig. 8G, H) altered the shape of stomatal cells and ruptured the guard cells. Nanofullerene C60 (Fig. 8A, B) caused a change in stomatal cells, but with less intensity than the nanotubes. Application of GNPs (Fig. 8D, E) caused even smaller changes. Sections C, F, and I of Fig. 8 show the deposition of nanoparticles on the surface of stomatal cells.

## Discussion

In this study, the application of different concentrations of GNPs showed a significant increase in the number of flowers per plant when compared to the control group (no application of nanoparticles and 100% FC irrigation) under irrigation conditions of 50% of the agricultural capacity. However, other carbon nanoparticles did not have the same effect. Carbon-based nanomaterials have been found to have the ability to promote early flower growth and increase flower and fruit production in plants grown under hydroponic conditions or soil exposed to carbon-based nanomaterials. For instance, in one study, the application of GNPs increased the number of flowers by 58% under salinity stress [31, 32]. In the present study, the application of carbon nanoparticles helped to moderate the effect of water deficit stress on the characteristics of wet and dry weight of roots. The effects observed in CNMs-treated plants are similar to the shade avoidance response (SAR) of Arabidopsis, such as an increase in stem length, root length, root number, cotyledon area, chlorophyll content and total sugar content. The SAR phenotype in CNMs-treated plants may be regulated by jasmonic acid, gibberellic acid, and auxin pathway components [33]. In another study investigating the effect of carbon nanoparticles on mung beans, moderate concentrations of carbon nanoparticles (100 to 150 µmol) resulted in an increase in total chlorophyll content (1.9 times), protein content (1.14 times), and plant biomass (fresh weight: 1.2 times, dry weight: 1.14 times), promoting the growth of treated plants [34].

The study found that using carbon nanoparticles did not alleviate the negative impact of stress on fruit number reduction. However, applying 200 mg/L of GNPs and 1000 mg/L of multi-walled nanotubes during 100% FC irrigation significantly increased the number of fruits compared to no application of nano at the same irrigation level. Exposure to CNMs also boosted tomato production by 200% [35], which is in line with the results of this study. The findings suggest that GNPs and nanofluorene C60 were more effective in increasing the fresh and dry fruit weight than multi-walled nanotubes. This difference indicates that the size, shape, and carbon nature of nanoparticles play a role in the results obtained. The impact of carbon nanoparticles depends on the exposure conditions, type of nanoparticle, dispersion state, and concentration [36]. CNMs mitigated the negative effect of stress on fruit length and diameter. Moreover, exposure to CNMs increased plant tissue size by affecting all three main signaling pathways of photosensitive receptors [37]. Although there was a significant difference between the effects of applied concentrations on the ion leakage index in this study, the applied carbon nanoparticles did not significantly reduce the effect of stress on this attribute. The concentration of 1000 mg/L of MWNTs had a significant effect in increasing the rate of photosynthesis and stomatal conductance in both stress and non-stress conditions.

**Fig. 9:**
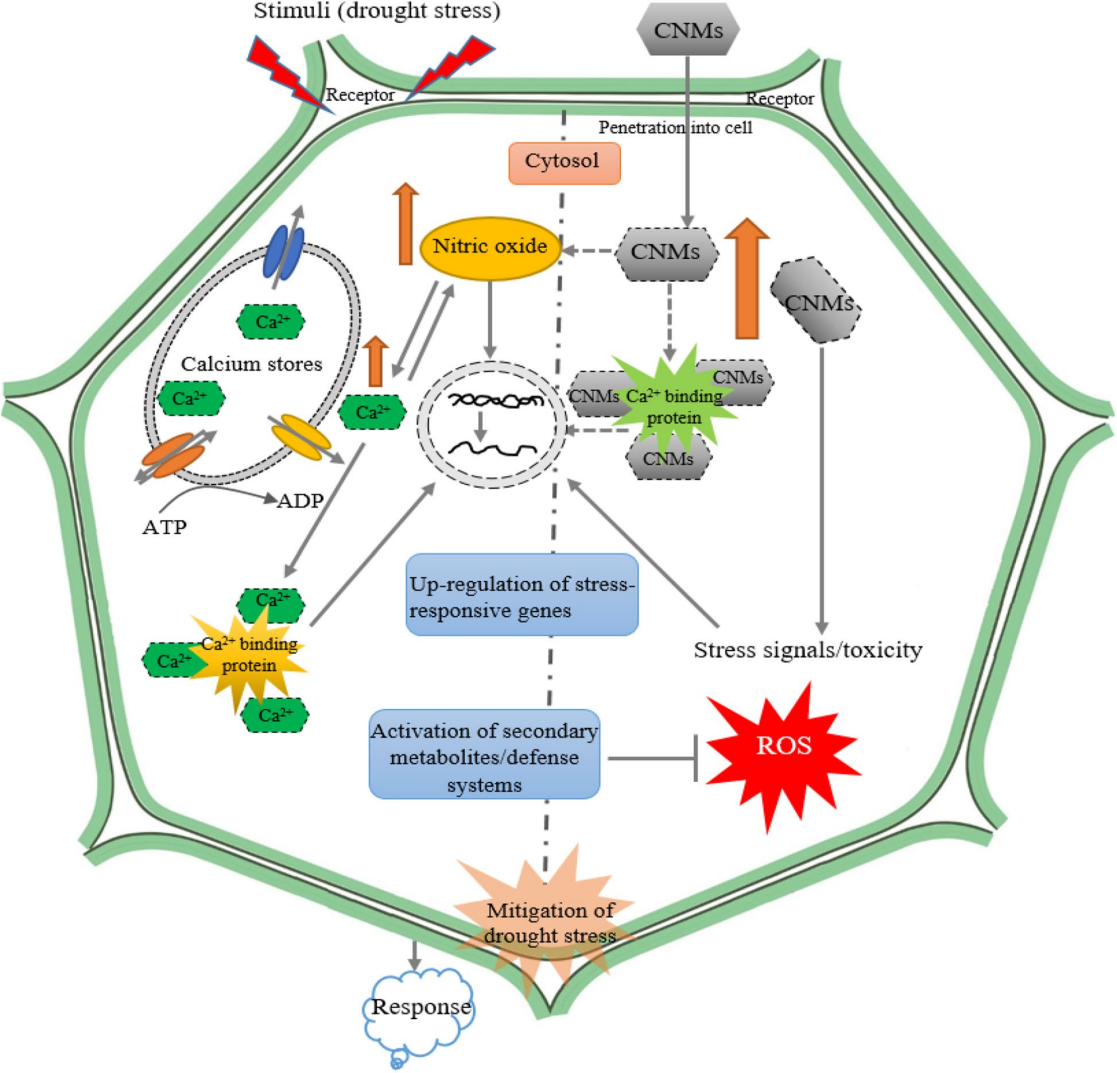
A schematic model about the potential role of carbon nanomaterials (CNMs) in plant cells under drought stress. This model includes various signaling pathways that are activated by CNMs, such as the up-regulation of defense mechanisms, redox regulatory and antioxidant systems, expression of drought-responsive genes, and biosynthesis of secondary metabolites and phytohormones. When plants are under drought stress, it leads to an increase in cytosolic Ca^2+^ level and the accumulation of reactive oxygen species (ROS) in cells, which causes oxidative stress. As a result, ROS can alter the macromolecules in the cytoplasm and degrade the cell membrane. This can also lead to a decrease in photosynthetic pigments content, ultimately reducing the photosynthetic activity of the plant. If prolonged, oxidative stress can ultimately lead to cell death. However, when plants are treated with CNMs, they can interact with elicitor/receptor-binding sites at the surface of the cell membrane, then enter the cell through different ways and form a complex with transporter ions. This leads to the over-expression of Ca^2+^ binding proteins, which can regulate several complex signaling phenomena. These include the accumulation of osmoprotectants, the improvement of the activity of antioxidants and MAPK cascades, the increase in biosynthesis of hormones such as nitric oxide, and the activation of gene-specific transcription factors [43–45]. Studies have shown that CNMs can play an important role in mitigating the effects of drought stress on plants

The application of carbon nanoparticles on corn significantly increased the plants height by 21.4%, as well as the dry biomass of the shoots and roots by 27.1% and 56.6%, respectively. Additionally, the absorption of nutrients such as nitrogen, phosphorus, potassium, calcium, magnesium, iron, manganese, copper, and zinc was increased by 133%, 41%, 192%, 209%, 106%, 59.6%, 155%, 105%, and 117% respectively [38]. Furthermore, the use of carbon nanoparticles also improved the photosynthetic parameters, chemical, and biochemical properties of the soil. Another study examined the effects of using different concentrations of graphene on the plant's chlorophyll b content under water stress [20]. The study found that the use of 1000 mg/L of nanofullerene, 200 mg/L of graphene, and 100 mg/L of multi-walled nanotubes significantly increased the amount of chlorophyll b compared to the control group (no use of nanoparticles in 100% FC irrigation). However, the intensity of the effect of water stress on the amount of chlorophyll b was significantly increased with the use of graphene. In another experiment, the effects of different concentrations of nanofullerene on the Feverfew content of Chamomile plant were compared. The study found that the highest amount of Feverfew content (23.6% more than the control group) was related to the foliar spraying of 1000 mg/L of fullerene. The use of carbon nanoparticles also increased the amount of total phenol, total flavonoid, and antioxidant activity. The concentrations of 100 and 1000 mg/L of fullerene and 200 and 1000 mg/L of GNPs were found to be more effective in increasing the amount of total phenol. The concentrations of 100 mg/L of fullerene and 1000 mg/L of GNPs were more effective in increasing the amount of total flavonoids. The concentrations of 1000 mg/L of nanofullerene and 200 mg/L of multi-walled nanotubes were more effective in terms of antioxidant activity [39]. The study also found that carbon nanoparticles caused changes in stomatal cells, which were more visible in the application of nanotubes. The deposition of fullerene nanoparticles on the stomatal cell surface was more than other nanoparticles [40]. Carbon-based nanomaterials enter the plant cell wall in the form of clusters with a filamentous structure on the surface of cells and lead to changes in metabolic processes [41]. SEM images showed a wider deposition of fullerene C60 on the leaf tissue of Feverfew variety Pharmasaat exposed to high concentration, which includes changes in trichome density and tissue tearing. The exact mechanism underlying interspecies variation in NPs uptake and drought stress amelioration in plants is still under investigation [42]. However, a schematic model has been proposed regarding the potential role of CNMs in plant cells under drought stress conditions (Fig. 9).

## Conclusions

The study found that applying concentrations of 100, 200 and 1000 mg/L of GNPs increased the number of flowers per plant when the plants were irrigated with 50% FC, compared to the control group which received no nanomaterials upon 100% FC irrigation. Other CNMs did not show significant effect on plant performance. Additionally, applying CNMs to the leaves helped to mitigate the deleterious effects of water deficit stress on root fresh and dry weight traits, though it did not have an impact on reducing the number of fruits affected by stress. However, applying 200 mg/L of GNPs and 1000 mg/L of MWNTs under 100% FC irrigation increased the number of fruits compared to non-application of nanomaterials with the same level of irrigation. The applied CNMs did not significantly diminish the effect of drought stress on the ion leakage index, despite there being a significant difference between the effects of applied concentrations on this attribute. A concentration of 1000 mg/L of MWNTs had a significant effect on increasing the rate of photosynthesis and stomatal conductance, in both stress and non-stress conditions compared to control. The application of different concentrations of GNPs increased the intensity of water stress on the amount of chlorophyll b. However, under well-watered conditions, applying a concentration of 1000 mg/L of fullerene C60, 200 mg/L of GNPs, and 100 mg/L of MWNTs enhanced the amount of chlorophyll b compared to the control group. The use of CNMs increased the amount of total phenol, total flavonoid, and antioxidant activity. The SEM images showed that CNMs caused changes in the stomatal cells, which were more visible in the case of nanotubes. The study demonstrated that the effect of CNMs on plant traits depends on the type and concentration of the nanoparticles applied. Further life cycle and mechanistic analyses are required to evaluate their regulatory effects of CNMs on gene expression involved in metabolic pathways to ensure safety and quality for nutraceutical products.

## Data Availability

The raw data of this article will be made available by corresponding author (Prof. Dr. Mansour Ghorbanpour; m-ghorbanpour@araku.ac.ir), according to the personal requests.
